# Normocomplementaemic Urticarial Vasculitis in a 19-Month-Old Girl

**DOI:** 10.1155/2016/1691290

**Published:** 2016-10-13

**Authors:** Peter Williams

**Affiliations:** Department of Anaesthesia and Pain Management, Royal Melbourne Hospital, Grattan Street, Parkville, VIC, Australia

## Abstract

Urticaria is common in children. Urticarial vasculitis (UV) is a potentially more serious, rare variant. The youngest reported case was 12 months of age. A systemically well, 19-month-old girl presented with her mother who was concerned about the development of a rash. On presentation, the child had normal vital signs, was alert, and was well and playing with toys. There was a widespread urticarial rash (raised, pruritic, and erythematous) that was most apparent on the trunk with minimal rash on the legs. Overlying this urticarial rash in a similar distribution was a blotchy, palpable purpuric rash and associated hyperpigmentation. Investigations revealed a normal level of haemoglobin, white cells, platelets, and electrolytes. Renal function, international normalised ratio, and activated partial thromboplastin time were all normal. There was no blood or protein in the urine. The erythrocyte sedimentation rate was mildly elevated at 19 mm/hour. Complement results (including C1q) obtained later were normal. This case is striking not only because of the rarity of UV in children but also due to the unique diagnostic and prognostic challenges that it raises.

## 1. Case

Urticaria is common in children. Urticarial vasculitis (UV) is a potentially more serious, rare variant with a peak incidence in the fourth decade of life [1]. The youngest reported case was 12 months of age [2]. UV is part of a spectrum from urticaria to vasculitis and must be differentiated from other causes of purpura and investigated with urine analysis and complement testing.

In regional Australia, a systemically well, 19-month-old girl presented with her mother who was concerned about the development of a rash. Three days earlier, the child developed a widespread pruritic, urticarial rash predominantly on the trunk. In the last two days, the mother became concerned when the urticarial rash turned purpuric.

**Figure 1:**
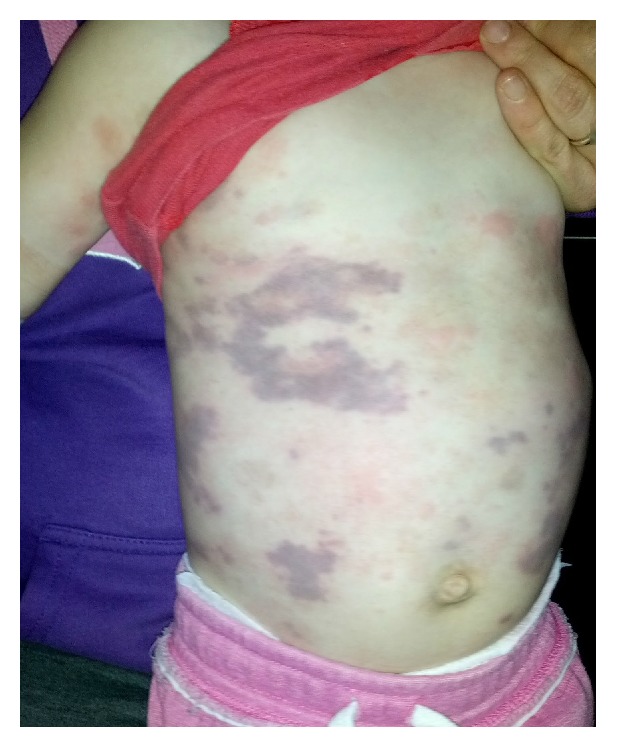
Widespread urticarial rash with overlying blotchy, palpable purpuric rash.

On presentation, the child had normal vital signs, was alert, and was well and playing with toys. On examination, there was an effervescent erythematous rash with associated angioedema which on resolution left a violaceous palpable purpuric rash that was reticulated, not well defined, and most apparent on the trunk with minimal rash on the legs (Figure 1). The child was not in pain and did not have arthralgia or abdominal discomfort. The child had been systemically well despite having minor coryzal symptoms five days earlier. She took no regular medications and in particular had taken no recent medications. The child was fully immunised according to the Australian immunisation schedule. No recent vaccines had been received. Her medical history included eczema. A family history of asthma and atopy was elicited.

Investigations revealed a normal level of haemoglobin, white cells, platelets, and electrolytes. Renal function, international normalised ratio, and activated partial thromboplastin time were all normal. There was no blood or protein in the urine. The erythrocyte sedimentation rate was mildly elevated at 19 mm/hour. Complement results (including C1q) obtained later were normal.

This case is striking not only because of the rarity of UV in children but also due to the unique diagnostic and prognostic challenges that it raises. UV is most common in females (60 to 80% of cases [3]) and the rash lasts longer than 24 hours. UV must be differentiated from idiopathic or immune-mediated thrombocytopenia (ITP). In ITP, platelets are low and the lesions are not usually palpable. Henoch-Schonlein purpura (HSP) is a form of vasculitis and associated with palpable purpura. In HSP, dermatologic findings may precede or follow systemic symptoms. HSP will classically present with the triad of arthralgia, abdominal pain, and purpura that predominately involves the buttocks and legs.

UV can classified into normocomplementaemic (NUV) and hypocomplementaemic (HUV) depending on levels of C1q. This has prognostic implications with the former being associated with a 2% incidence of systemic lupus erythematosus (SLE) versus 50% with the latter [1]. Renal involvement may be apparent and proteinuria and haematuria should be identified. Whilst UV has both a clinical and a histologic component, a skin biopsy was thought not to be necessary in the first instance by the treating clinician. A skin biopsy was thought to be warranted if it persisted or recurred.

This child was normocomplementaemic and was discharged with cetirizine oral drops (0.125 mg/kg). The rash was thought to be likely viral in aetiology. She was seen by a paediatrician six weeks later and was found to be well. Her rash had resolved and consequently no biopsy was performed. The child will continue to be periodically monitored as NUV is known to reoccur, complement levels may subsequently fall, and involvement of other organs may become apparent at a later stage.
